# Activity-stability trade-off observed in variants at position 315 of the GH10 xylanase XynR

**DOI:** 10.1038/s41598-024-57819-z

**Published:** 2024-04-02

**Authors:** Tomoka Nakamura, Teisuke Takita, Kohei Kuwata, Kimihiko Mizutani, Bunzo Mikami, Satoshi Nakamura, Kiyoshi Yasukawa

**Affiliations:** 1 Division of Food Science and Biotechnology, Graduate School of Agriculture, Kyoto University, Sakyo-ku, Kyoto, 606-8502 Japan; 2 Division of Applied Life Sciences, Graduate School of Agriculture, Kyoto University, Uji, Kyoto 611-0011 Japan; 3 Research Institute for Sustainable Humanosphere, Kyoto University, Uji, Kyoto 611-0011 Japan; 4 Institute of Advanced Energy, Kyoto University, Uji, Kyoto 611-0011 Japan; 5 Department of Life Science and Technology, Tokyo Institute of Technology, Midori-ku, Yokohama, 226-8501 Japan

**Keywords:** Biochemistry, Biotechnology

## Abstract

XynR is a thermostable alkaline GH10 xylanase, for which we have previously examined the effects of saturation mutagenesis at position 315 on enzyme alkaliphily, and found that at pH 10, the activities of variants could be ordered as follows: T315Q > T315S = T315N > T315H = wild-type XynR (WT) > 15 other variants. In this study, we sought to elucidate the mechanisms underlying the variable activity of these different variants. Crystallographic analysis revealed that the Ca^2+^ ion near position 315 in WT was absent in the T315Q variant. We accordingly hypothesized that the enhancement of alkaliphily in T315Q, and probably also in the T315H, T315N, and T315S variants, could be ascribed to an activity-stability trade-off associated with a reduction in stability due to the lack of this Ca^2+^ ion. Consistent with expectations, the alkaline resistance of T315H, T315N, T315Q, and T315S, evaluated through the pH-dependence of stability at 0 mM CaCl_2_ under alkaline conditions, was found to be lower than that of WT: the residual activity at pH 11 of WT was 78% while those of T315H, T315N, T315Q, and T315S were 0, 9, 0, and 43%, respectively. In addition, the thermostabilities of these four variants, as assessed using the denaturing temperatures (*T*_m_) at 0 mM CaCl_2_ based on ellipticity at 222 nm in circular dichroism measurements, were lower than that of WT by 2–8 °C. Furthermore, the *T*_m_ values of WT and variants at 5 mM CaCl_2_ were higher than those at 0 mM CaCl_2_ by 6–11 °C. Collectively, our findings in this study indicate that mutation of the T residue at position 315 of XynR to H, N, Q, and S causes an increase in the alkaliphily of this enzyme, thereby reducing its stability.

## Introduction

Xylanase [EC 3.2.1.8] hydrolyzes the internal β-1,4-linkage of xylan. Xylanase is widely used in food, paper and pulp, and biofuel industries^1–3^. In such industries, xylanase with high activity at high pH conditions is highly desirable^4^. Most xylanases are grouped in the family of glycoside hydrolase (GH) 10 or 11. GH10 and GH11 xylanases have two conserved catalytically important E residues in the active site. However, there is no sequence or structural similarity between GH10 and GH11 xylanases. GH10 xylanases have a (β/α)_8_ triose-phosphate isomerase (TIM) barrel fold while GH11 xylanases have a β-jellyroll fold composed of eight β strands^5^. GH10 xylanases have a shallow active site while GH11 xylanases have a deep active site. Compared with GH11 xylanases, GH10 xylanases have lower substrate specificity and higher thermostability^6^.

Increase in alkaliphily has been an important protein engineering research target. For this purpose, various strategies have been so far applied, such as introduction of multiple Arg on protein surface^7^, random mutagenesis^8^, and substitution of amino acid residues in the active site and on protein surface^9^. TAR-1 is a thermophilic and alkaliphilic *Bacillus* sp. strain isolated from a soil sample from Kanagawa, Japan^10^. A GH10 xylanase XynR was identified in the culture broth of TAR-1^10^. We selected T315N as an alkaliphilic XynR variant from a site saturation mutagenesis library^11^. Recently we examined the effects of amino acid residue at position 315 of XynR on its alkaliphily, showing that T315H, T315Q, and T315S exhibited high alkaliphily in the hydrolysis of beechwood xylan^12^. The activities of T315H, T315N, T315Q, and T315S at pH 9.0 and 10.0 were 100–130% of WT (Fig. S1)^12^, and the reducing sugars increased with increasing the reaction time (0–2 h)^12^. In this study, we explored the mechanism of alkaliphily in these variants.

## Results

### Refinement of the structure of T315Q

**Figure 1:**
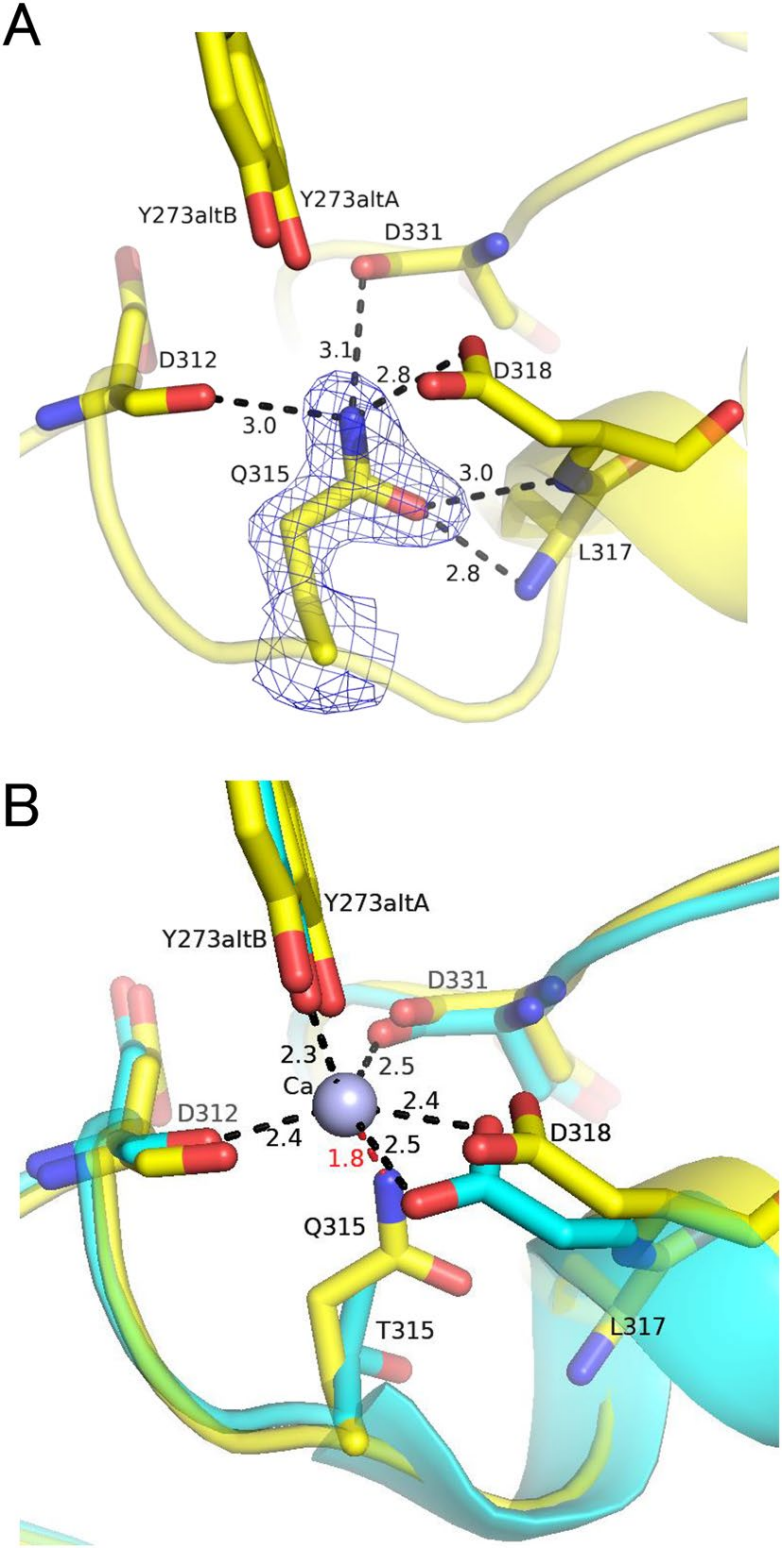
Ca^2+^-binding site in the active site. (**A**) T315Q. Phenix polder omit of Q315 is contoured at 4σ. (**B**) Superposition of T315Q and WT. The peptides of T315Q and WT are colored in yellow and cyan, respectively. The Ca^2+^ is shown as a grey sphere. The number indicates the distance (Å).

We made crystallographic analysis of T315Q. Table 1 summarizes data collection and refinement statistics. The space group of the crystal was *C2*. The structure was refined at 1.90 Å resolution. The whole structure of T315Q exhibited no difference compared to WT^13^ with rmsd of 0.65 Å for 353 Cα atoms. Figure 1A shows the mutation-site structure of T315Q. Phenix polder omit map shows that T315 was replaced with Q in T315Q. WT had three Ca^2+^ ions^13^, but T315Q had one Ca^2+^ ion. Figure 1B shows the comparison of mutation-site structures of WT and T315Q. In WT, the Ca^2+^ ion was observed, which was surrounded by four ligating residues (D312, D318, D331, and Y273), suggesting that this Ca^2+^ ion plays certain roles in the stability of active site. T315Q lacked this Ca^2+^ ion. In WT, T315 did not interact with the Ca^2+^ ion. In T315Q, the side chain of Q315 protrudes inside, resulting in that the distance of Nε2 from the position where the Ca^2+^ ion located in WT was 1.8 Å. This suggested that such unfavorable distance (1.8 Å) leaded to the steric hindrance between the Nε2 of Q315 and the Ca^2+^ ion, resulting in the lack of the Ca^2+^ ion in T315Q. The Nε2 of Q315 forms hydrogen bonds with Oδ1 of D318, O of D331 and O of D312 instead of the coordination bond of Ca^2+^ ion in WT, suggesting partial stabilization of this site.

**Table 1:** Data collection and refinement statistics of XynR.

	T315Q	WT/xylobiose
A. Diffraction data		
X-ray source	SPring-8/BL26B1	SPring-8/BL26B1
Detector	DECTRIS EIGER 4 M	DECTRIS EIGER 4 M
Wavelength (Å)	1.0	1.0
Resolution range (Å)	50.00–1.90 (2.01–1.90)	50.00–1.80 (1.90–1.80)
Space group	*C2*	*P2*_*1*_
Unit cell parameters		
a, b, c (Å), β (º)	105.179, 41.783, 80.873, 90.523	88.702, 53.335, 96.567
Unique reflections	26,490 (3206)	82,572 (12,885)
Multiplicity	3.87 (2.46)	3.4 (3.4)
Completeness (%)	94.4 (71.3)	97.5 (94.8)
Mean *I*/σ (*I*)	13.1 (2.28)	16.8 (2.49)
Wilson *B*-factor (Å^2^)	29.19	31.90
*R*_merge_ (%)	6.8 (34.8)	4.6 (47.6)
*R*_meas_ (%)	7.8 (43.4)	5.4 (56.5)
CC_1/2_ (%)	99.8 (82.9)	99.9 (84.6)
B. Refinement statistics		
Resolution range used refinement	44.3–1.90 (1.98–1.90)	44.3–1.80 (1.82–1.80)
*R*_work_ (%)	16.2 (24.7)	16.6 (32.8)
*R*_free_ (%)	20.6 (29.0)	20.2 (37.7)
Number of residues		
Protein residurs	353 (1–353)	716 (2–359 × 2)
Ca^2+^/I/PEG/MPD/TRS/ACT/XYB/water	1/4/4/0/0/0/0/335	9/0/2/7/3/4/8/551
R.m.s.d., bond lengths (Å)	0.008	0.007
R.m.s.d., bond angles (°)	0.940	0.865
Ramachandran favored (%)	97.7	97.6
Ramachandran outliers (%)	0	0
Rotamer outliers (%)	0.65	1.08
Clash score	3.14	4.18
PDB ID	8XY0	8Y1M

Generally, mutations which increase activity are accompanied with decrease in stability and vice versa. This relationship between activity and stability was well known as activity-stability trade-offs^14,15^. To address this hypothesis, we compared the stabilities of WT and variants in the subsequent studies.

### pH dependence of stability

**Figure 2:**
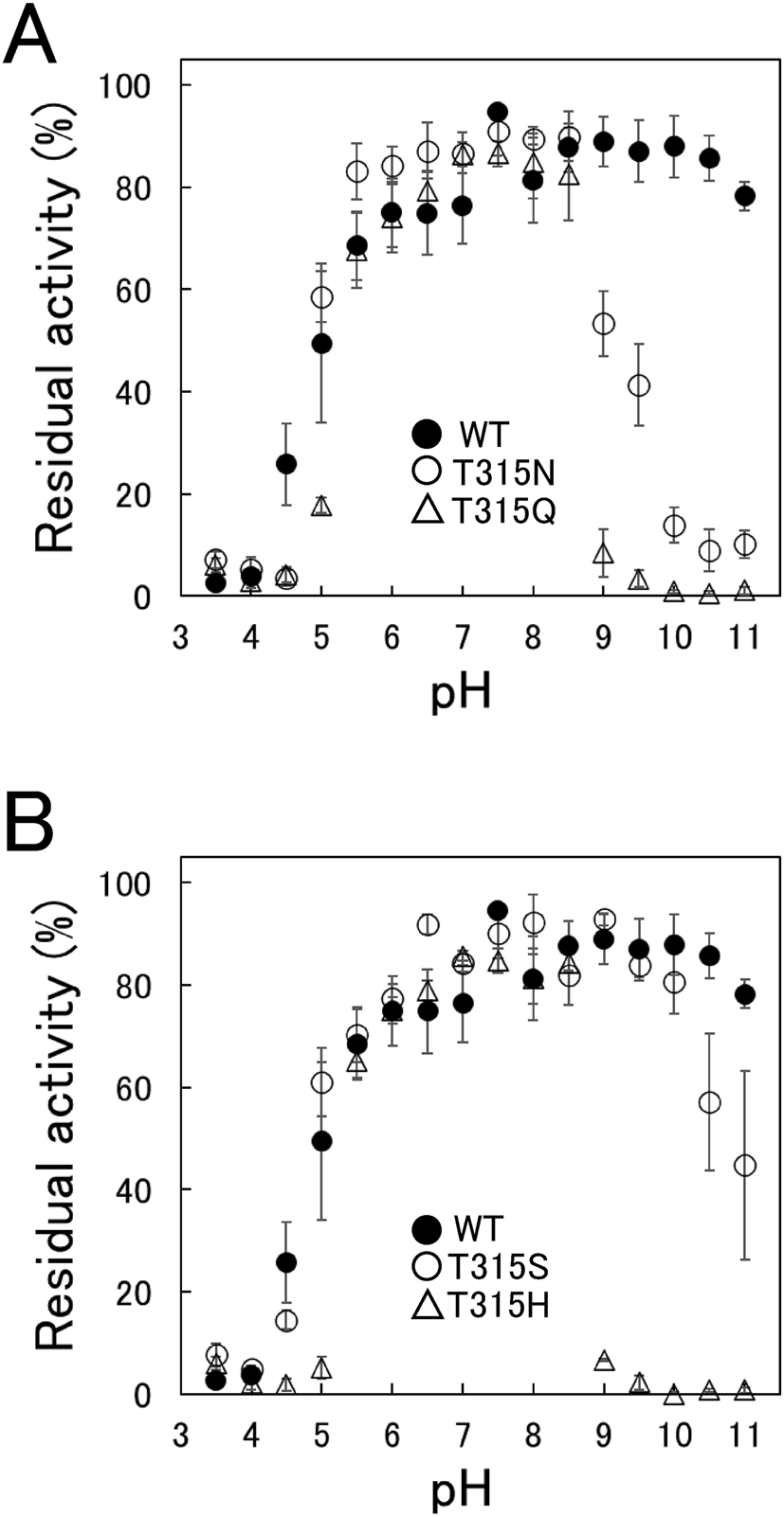
Effect of pH on stabilities of WT and variants. The enzymes (0.15 μM) were incubated at various pHs and 37 °C for 24 h. The incubation buffers were 100 mM acetate-sodium acetate buffer (pH 3.5–5.5), 100 mM phosphate-NaOH buffer (pH 6.0–8.5), and 100 mM carbonate-bicarbonate buffer (pH 9.0–11.0). Then, hydrolysis reaction of beechwood xylan was carried out at 37 °C with the enzyme and initial substrate concentrations of 0.015 μM and 9 mg/mL, respectively. Residual activity indicates the value compared to that before the incubation at pH 3.5–11.0. Error bars indicate SD values of triplicate determinations.

We examined pH dependences of stability of WT and four variants (T315H, T315N, T315Q, and T315S). The enzymes were incubated at various pHs and 37 °C for 24 h. After incubation, the activities to hydrolyze beechwood xylan were measured at pH 8.0 and 37 °C (Fig. 2). T315Q and T315H exhibited markedly narrower bell-shaped pH-dependence of stabilities than WT. T315N and T315S also exhibited narrower bell-shaped pH-dependences of stability than WT. The residual activity at pH 11 of WT was 78% while those of T315H, T315N, T315Q, and T315S were 0, 9, 0, and 43%, respectively. These results suggested that the amino acid residue whose side chain has an amido or imidazole group at position 315 makes XynR less alkaline-resistant.

### Thermal denaturation

Scheme 1 has been used for evaluating enzyme stability.

Scheme 1 $$N \rightleftarrows D$$

where *N* and *D* represent the native and denatured species, respectively. In Scheme 1, the stability of protein is assessed by Δ*G°* that represents the difference in *G°* between the native and denatured states at a certain temperature or *T*_m_, the temperature at which *F*_u_ is 0.5 in Eq. (1).

**Figure 3:**
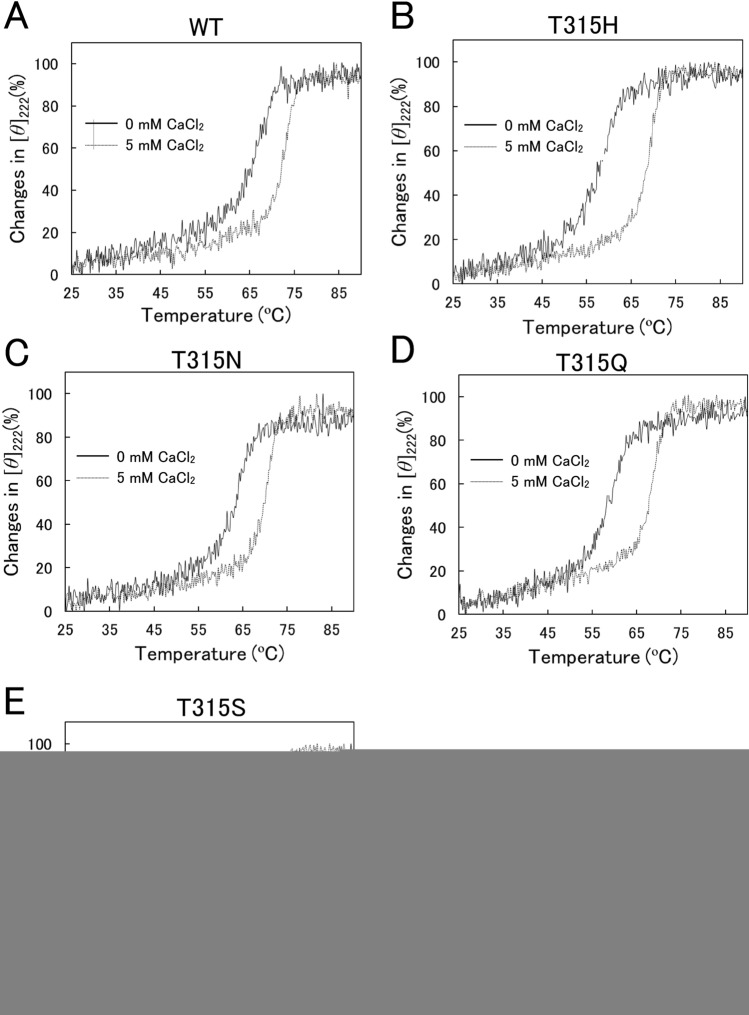
CD analyses of WT and variants. Changes in [*θ*]_222_ of the enzymes were monitored in 20 mM phosphate-NaOH buffer (pH 8.0) containing 0 or 5 mM CaCl_2_ from 25 to 90 °C at 0.5 °C/min.

The CD spectra of WT and four variants (T315H, T315N, T315Q, and T315S) at pH 8.0 and 25 °C exhibited negative ellipticities at around 200–250 nm with minimum values around 222 nm^12^, indicating the mutation at position 315 did not elicit drastic structural changes. Figure 3A–E showed thermal denaturation of WT and variants by monitoring [*θ*]_222_ in the range of 25–90 °C. The denaturation curves of WT and variants showed apparent two-state model. However, this thermal denaturation was not reversible, suggesting that Scheme 1 was not fully applicable. The *T*_m_ values at 0 and 5 mM CaCl_2_ of WT and variants are shown in Table 2. They were in the order of WT > T315N ≈ T315S > T315H ≈ T315Q, indicating that the mutation at position 315 decreased the stability of XynR. The *T*_m_ values at 5 mM CaCl_2_ were 6.4–10.6 °C higher than those at 0 mM CaCl_2_. This suggested that thermal treatment promotes the dissociation of the Ca^2+^ ion from the active site, while addition of excess Ca^2+^ ions promotes the binding.

**Table 2:** *T*_m_ of WT and variants.

	*T*_m_ (°C)		B–A (°C)
	0 mM CaCl_2_ (A)	5 mM CaCl_2_ (B)	
WT	65.0	71.9	6.9
T315H	57.5 (− 7.5)	68.1 (− 3.8)	10.6 (+ 3.7)
T315N	63.2 (− 1.8)	69.6 (− 2.3)	6.4 (− 0.5)
T315Q	58.0 (− 7.0)	67.5 (− 4.4)	9.5 (+ 2.6)
T315S	63.3 (− 1.7)	70.1 (− 1.8)	6.8 (− 0.1)

Averages of duplicate determinations are shown. Values in parentheses indicate those relative to WT.

It is reported that the molten globule state is an intermediate state that has lost the majority of tertiary structure but retains a significant amount of secondary structures^16^, suggesting that the *T*_m_ values might be lower than those in Table 2 if the values were assessed by the other methods such as fluorescence and differential scanning calorimetry.

### Thermal inactivation

Scheme 2 has also been used for evaluating enzyme stability.

Scheme 2 $$N \rightleftarrows PD \to D$$

where *N*, *D*, and *PD* represent the native, denatured, and partially denatured species, respectively. In Scheme 2, stability of protein is assessed by Δ*G*°^‡^ that represents the difference in *G*°^‡^ between the native and transition states and is obtained from the *k*_obs_ using Eq. (3).

**Figure 4:**
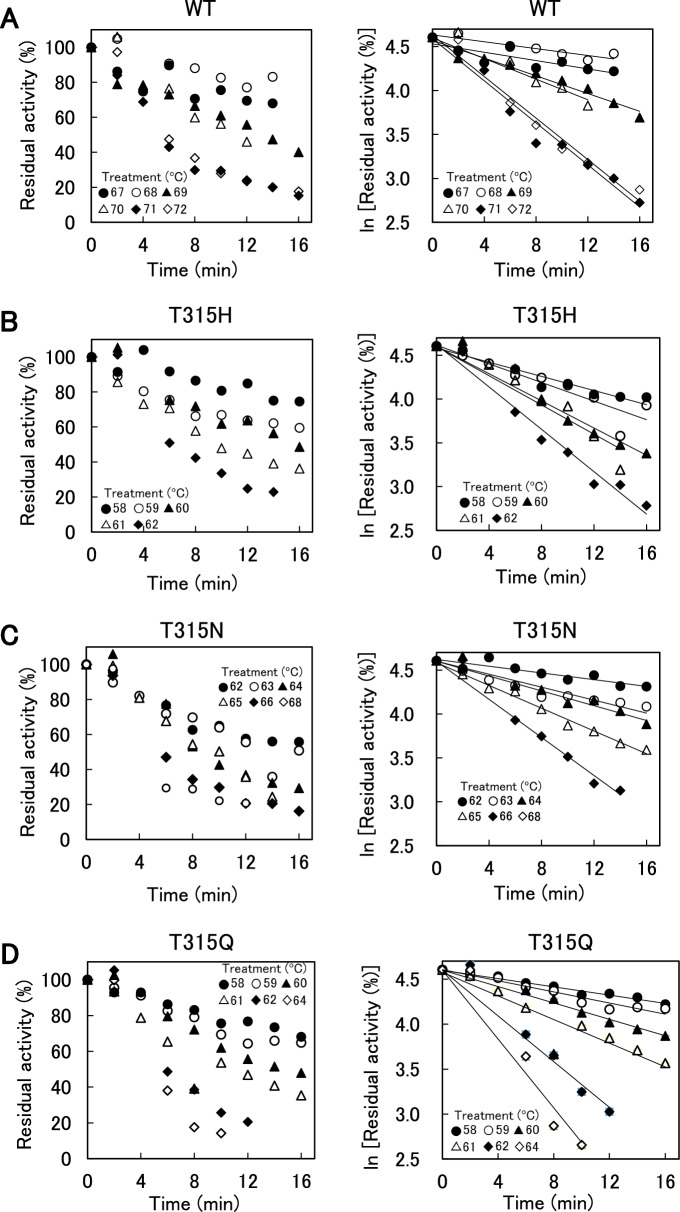

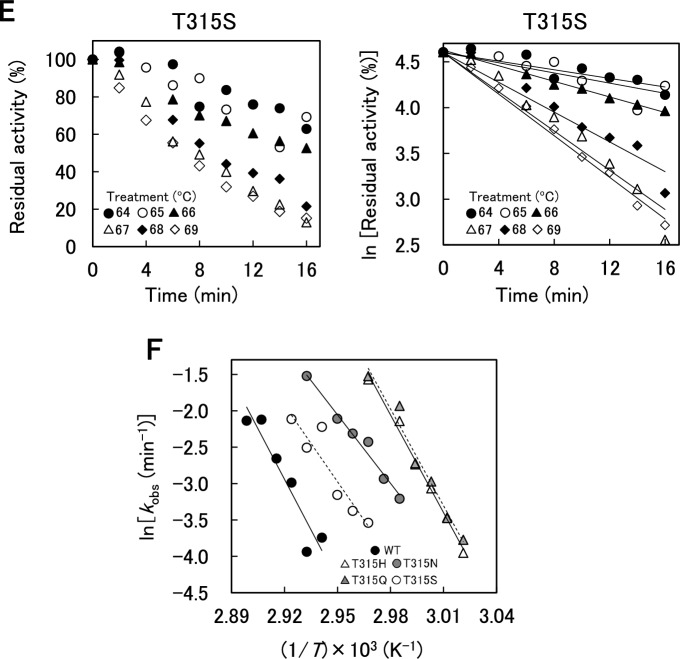
Thermal inactivation of WT and variants. (**A–E**) The enzymes (1.5 μM) were incubated in 100 mM HEPES–NaOH buffer (pH 8.0) at 58–72 °C for specified time. Beechwood xylan-hydrolysis activities were measured at 37 °C after thermal incubation. Residual activity was expressed as the relative value to that before the incubation at 58–72 °C. Residual activities were plotted against incubation time. (**F**) Arrhenius plot of *k*_obs_ values. The natural logarithms of *k*_obs_ values were plotted against the reciprocal of absolute temperature of thermal inactivation.

WT and variants were incubated at pH 8.0 and 58–72 °C for 2–16 min, and the remaining activities were determined at pH 8.0 and 37 °C (Fig. 4A–E, left panel). The natural logarithm of the remaining activities of WT and variants plotted against the incubation time gave linear relationships at all temperatures examined (Fig. 4A–E, right panel), indicating that the inactivation followed pseudo-first-order kinetics. The *k*_obs_ values and half-life (*τ*_1/2_) values of WT and variants at each temperature are summarized in Table S1. They increased with increasing temperatures. The temperatures at which the *k*_obs_ values were 0.05–0.10 min^–1^ in the order of WT > T315N ≈ T315S > T315H ≈ T315Q, indicating again that the mutation at position 315 decreased the stability of XynR.

Figure 4F shows the Arrhenius plot of *k*_obs_. Linear relationship was obtained between the logarithmic value of* k*_obs_ and 1/*T*. The thermodynamic parameters for thermal inactivation of WT and variants at 65 °C, which are obtained using Eqs. (3)–(5), are summarized in Table 3. The Δ*G*°^‡^ values that reflect the protein stability were in the order of WT > T315N ≈ T315S > T315H ≈ T315Q. All variants exhibited decreased Δ*H*°^‡^ and Δ*S*°^‡^ values, indicating that the decreased thermal stability was due to the decrease in *∆H*°^‡^. Notably, the magnitude of decrease in Δ*H*°^‡^ and Δ*S*°^‡^ was marked in T315N and T315S but was slight in T315H and T315Q, indicating qualitative difference between the effects of mutation of T315 to N or S and that to H or Q.

**Table 3:** Thermodynamic parameters for thermal inactivation of WT and variants at 65 °C.

XynR	*E*_a_ (kJ mol^−1^)	Δ*G*°^‡^ (kJ mol^−1^)	Δ*H*°^‡^ (kJ mol^−1^)	Δ*S*°^‡^ (J mol^−1^ K^−1^)
WT	384 ± 131	92 ± 1	382 ± 131	857 ± 387
T315H	371 ± 20	82 ± 1	369 ± 20	849 ± 59
T315N	260 ± 41	86 ± 1	258 ± 41	510 ± 121
T315Q	368 ± 61	82 ± 1	366 ± 61	839 ± 181
T315S	292 ± 67	88 ± 1	289 ± 67	594 ± 199

*E*_a_, Δ*G*°^‡^, Δ*H*°^‡^, and Δ*S*°^‡^ are the activation energy, the Gibbs energy change of activation, the enthalpy change of activation, and the entropy change of activation, respectively. The average of triplicate determination with SD values is shown.

### Effects of Ca^2+^ binding on stability

**Figure 5:**
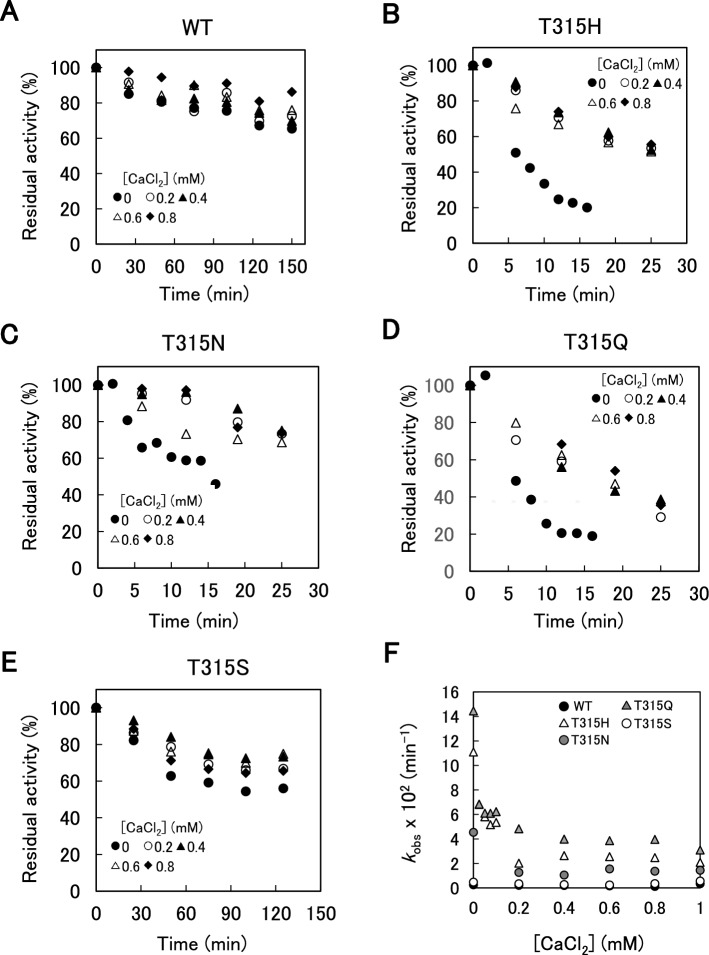
Effect of CaCl_2_ concentrations on thermal inactivation of WT and variants. The enzymes (1.5 μM) were incubated in 100 mM HEPES–NaOH buffer (pH 8.0) and 0–0.8 mM CaCl_2_ at 62 °C for specified time. Beechwood xylan-hydrolysis activities were measured at 37 °C after thermal incubation. (**A–E**) Residual activities of WT and four variants were plotted against incubation time. (**F**) First-order rate constants of thermal inactivation (*k*_obs_) of WT and four variants were plotted against CaCl_2_ concentrations of 0–1 mM.

To explore the role of the Ca^2+^ ion on the mutational effects at position 315, the thermal inactivation of WT and variants at 62 °C in the presence of various CaCl_2_ concentrations (0–5 mM) was investigated (Fig. 5, Fig. S2). The *k*_obs_ values are shown in Fig. 5F and summarized in Table S2. The *k*_obs_ values of WT and T315S were constant at all CaCl_2_ concentrations examined. The *k*_obs_ values of T315N were constant at all CaCl_2_ concentrations except for 0 mM. The *k*_obs_ values of T315H and T315Q decreased and their *τ*_1/2_ values increased with increasing CaCl_2_ concentrations (0–0.1 mM) and were constant at 0.1–5 mM CaCl_2_. The CaCl_2_ concentration-independent *k*_obs_ values (min^−1^) were 0.001–0.004 for WT, 0.020–0.054 for T315H, 0.007–0.016 for T315N, 0.029–0.062 for T315Q, and 0.0024–0.0086 for T315S (Table S2), indicating that the value was in the order of WT < T315S < T315N < T315H < T315Q.

## Discussion

Generally, mutations which increase activity are accompanied with decrease in stability and vice versa. This relationship between activity and stability was well known as activity-stability trade-offs^14,15^. In the previous study^12^, the alkaliphily was in the order of T315H ≈ T315Q > T315N ≈ T315S > WT. In this study, the alkaline resistance (Fig. 2), thermostability (Figs. 3, 4), and the Ca^2+^ binding ability (Fig. 5) were in the order of T315H ≈ T315Q < T315N ≈ T315S < WT, supporting for activity-stability trade-off.

**Figure 6:**
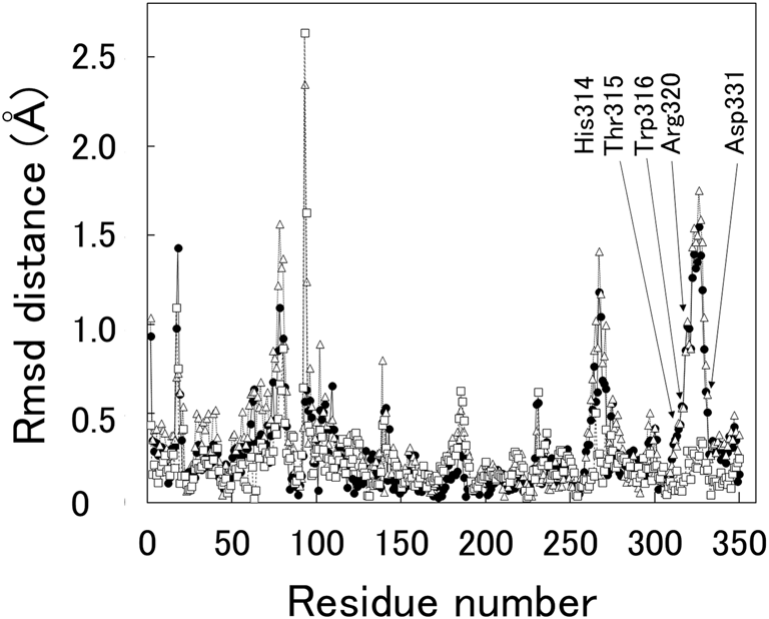
rmsd distance plot after superposition of WT (PDB 7CPK), T315Q (PDB 8XY0), and XynR complexed with xylobiose (PDB 8Y1M). Note that four peak regions corresponding to residues 15–20, 75–83, 105–109 and 264–267, respectively, are formed by the crystal packing effects and that the largest deviation occurs only in one peak region corresponding to residue 311–331. Symbols: T315Q-WT, closed circle; T315Q-XynR complexed with xylobiose, open triangle; WT-XynR complexed with xylobiose, open square.

Figure 6 shows the rmsd distance plot after superposition of the structures of T315Q (this study) and WT^13^. Five peaks appeared (15–20, 75–83, 105–109, 264–267, and 311–331). It should be noted that the 4 peak regions with residues 15–20, 75–83, 105–109, and 264–267 are formed by the crystal packing effects. Therefore, this result shows that the largest deviation occurs in the residues 311–331, which contains the Ca^2+^ binding site in the α helix (317–320) and the extended loop (321–331)^13^.

**Figure 7:**
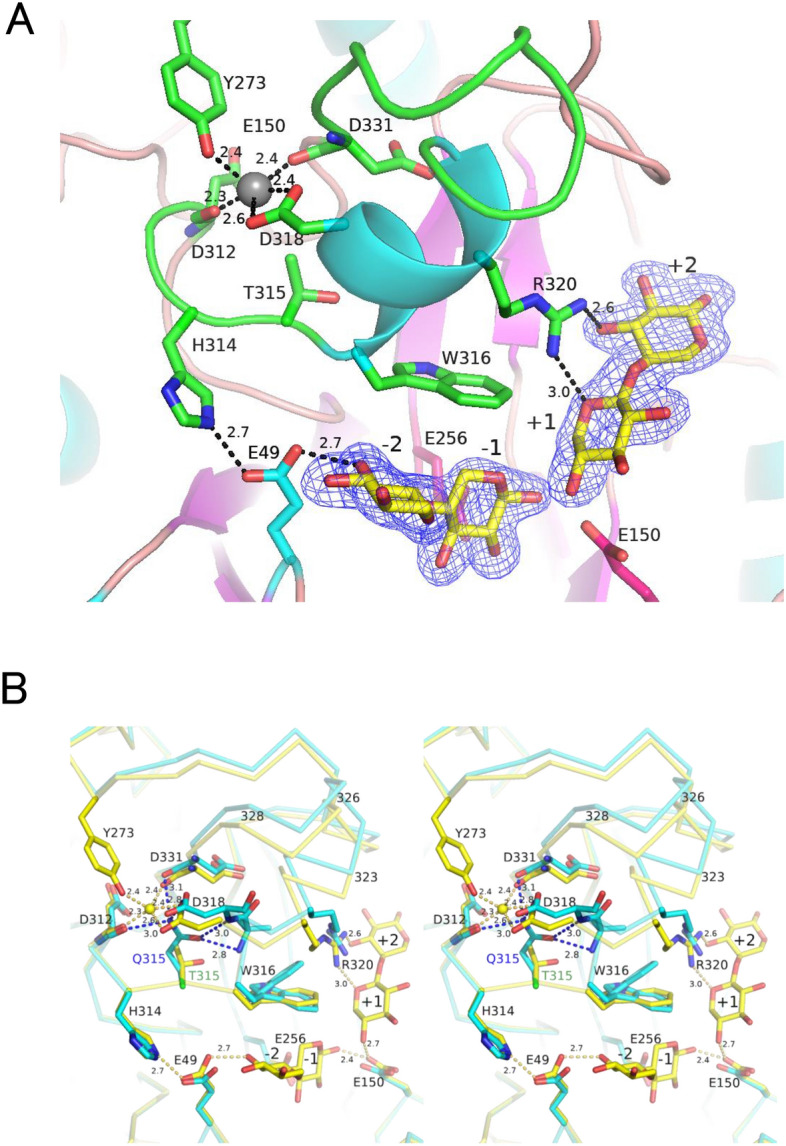
Active-site structure of XynR complexed with xylobiose. (**A**) WT. The peptide is colored in green, the xylobiose is colored in yellow, and the Ca^2+^ is shown as grey sphere. Phenix polder omit of xylobiose is contoured at 4σ. (**B**) Superposition of WT and T315Q (left and right stereo drawing). The peptides of WT and T315Q are colored in yellow and cyan, respectively. The Ca^2+^ is shown as yellow sphere. The number indicates the distance (Å).

To explore the role of this extended loop on the substrate binding and catalytic activity, we made crystallographic analysis of WT complexed with xylobiose. As shown in Table 1, the space group of the crystal was *P2*_1_, and the structure was refined at 1.80 Å resolution. The whole structure of WT complexed with xylobiose exhibited no difference compared to ligand-free WT^13^ with rmsd of 0.21 Å for 353 Cα atoms in chain A. Figure 7A shows the active-site structure of WT complexed with xylobiose, and Fig. 7B shows the comparison of WT and T315Q. In the region (residue 311–331) with the largest deviation between T315Q and WT (Fig. 6), three residues (D312, D318, and D331) are involved in the Ca^2+^ binding, and two residues are involved in the binding to xylobiose. R320 interacts with O5 of subsite + 1 xylose and O3 of subsite + 2^17^, and is located near the acidic and basic catalytic residues (E150 and E256, respectively) with 8.0 and 8.8 Å, respectively. These evidences suggest that the mutation of T315 to Q deviates the position of R320, affecting the dissociation of E150, leading to increase in alkaliphily of T315Q.

In conclusion, the alkaliphilic XynR variant T315Q lacked the Ca^2+^ ion near position 315 by keeping the interaction with three of four ligating residues with the Ca^2+^ ion. Our results suggested that T315Q, and presumably T315H, T315N, and T315S, obtained higher alkaliphily by decreasing stability accompanied by the loss of this Ca^2+^ ion and the resulting loop (residues 311–331) deviation.

## Methods

### Materials

pET-21b(+)-XynR (Fig. S3)^11,12^ was used as the expression plasmid for XynR in *Escherichia coli.* Beechwood xylan was purchased from Megazyme (Bray, Ireland). 3,5-Dinitrosalicylic acid (DNS) was purchased from Nacalai Tesque (Kyoto, Japan). The concentrations of WT and its variants, T315H, T315N, T315Q, and T315S, were determined using Protein Assay CBB Solution (Nacalai Tesque) with bovine serum albumin (Nacalai Tesque) as a standard.

### Expression and purification of WT and variants

One hundred milliliter of LB broth containing 50 μg/mL ampicillin was inoculated with the glycerol stock of the transformed *E. coli* BL21 (DE3) and incubated at 37 °C with shaking. When *OD*_660_ reached 0.6–0.8, isopropyl-β-D-thiogalactopyranoside (IPTG) (25 μL of 500 mM) was added, and growth was continued at 30 °C for 24 h. The cells were harvested by centrifugation and suspended with 50 mL of 20 mM phosphate-NaOH buffer (pH 8.0) (buffer A) and disrupted by sonication. After centrifugation, the supernatant was collected. Solid (NH_4_)_2_SO_4_ was added to the supernatant to be 50% saturation. The pellet resulted was collected by centrifugation, dissolved in buffer A containing 0.5 M NaCl (buffer B), and dialyzed against buffer A. The crude enzyme solution thus obtained was applied to a HisTrap™ HP column (GE Healthcare, Buckinghamshire, UK) equilibrated with buffer B. After the was wash with 50 mL of buffer B, XynR was recovered by the elution with buffer B containing 0.5 M imidazole. Each fraction (5 mL) was assessed to contain WT or variants by sodium dodecyl sulfate (SDS)-polyacrylamide gel electrophoresis (PAGE). Active fractions were concentrated after desalting. Purified enzyme solution was stored at 4 °C.

### Crystallization and structural determination

Crystals were obtained in the following conditions: (i) ligand-free T315Q: 1 μL of 12.7 mg/mL enzyme solution in 20 mM phosphate-NaOH buffer (pH 8.0) was mixed with 1 μL of reservoir solution (0.2 M ammonium iodide, 26% w/v polyethylene glycol (PEG) 4000, pH 5.9) and was equilibrated against 100 μL of reservoir solution at 20 °C, using the sitting drop vapor-diffusion method in a 96-well plate (Intelli-Plate, Art Robbins Instrument, Sunnyvale, CA). Rectangle crystals were obtained after a few weeks. (ii) WT complexed with xylobiose: 1 μL of 13.0 mg/mL enzyme solution in 0.35 M xylobiose and 20 mM tris-hydroxymethyl aminomethane (Tris)-HCl buffer (pH 8.0) was mixed with 1 μL of reservoir solution (0.02 M CaCl_2_, 0.1 M sodium acetate, 22% v/v 2-methyl-2,4-pentanediole, pH 4.6) and was equilibrated against 100 μL of reservoir solution at 20 °C, using the sitting drop vapor-diffusion method in a 96-well plate. Rectangle crystals were obtained after a few weeks. Crystals were soaked in the reservoir solution with 350 mM xylobiose.

Crystals were flash-cooled in nitrogen gas stream at 100 K. Diffraction data were collected at the BL26B1 station of SPring-8, Sayo, Hyogo, Japan with the approval of JASRI (proposal nos. 2021B2760 and 2023B2733), after the diffraction was checked by an in-house detector system of Bruker D8 Venture. The collected diffraction data were processed with XDS^18^. Molecular replacement was conducted using the structure of WT^13^ as a search model (PDB, 7CPK). Structure refinement was conducted using COOT^19^ and PHENIX^20^.

### Hydrolysis of beechwood xylan

The activity was measured as described previously^9,10^. Briefly, the reaction was initiated by mixing 10 μL of enzyme solution and 90 μL of substrate solution (10 mg/mL beechwood xylan in 100 mM phosphate-NaOH buffer at pH 8.0) both pre-incubated at 37 °C. The reaction solution was incubated at 37 °C, and 100 µL of DNS solution (0.5% w/v DNS, 1.6% w/v NaOH, 30% w/v potassium sodium tartrate) was added to stop the reaction at predetermined times. After incubating at 100 °C for 15 min and at 0 °C for 3 min, 80 µL of the solution and 120 µL of water were mixed, and *A*_540_ was measured with a multimodal plate reader EnSight (PerkinElmer, Waltham, MA). The standard curve was made using xylose. The concentrations of reducing sugars were estimated from the standard curve. The initial reaction rate was estimated from the time-course for production of reducing sugars.

### pH treatment

pH treatment of WT and variants was initiated by mixing 5 μL of enzyme solution (1.5 μM) and 45 μL of buffer solution (100 mM acetate-sodium acetate buffer (pH 3.5–5.5), 100 mM phosphate-NaOH buffer (pH 6.0–8.5), or 100 mM carbonate-bicarbonate buffer (pH 9.0–11.0)). The treatment continued at 37 °C for 24 h. Then, activity of the pH-treated enzyme solution was measured as described above.

### Circular dichroism (CD) measurement

The CD spectra of WT and variants were measured using a J-820 spectropolarimeter (Jasco, Tokyo, Japan) with a Peltier system of cell temperature control under the following conditions: spectral range 200–250 nm; 100 mdeg sensitivity; 0.2 nm resolutions; 1 s response time; 10 nm min^−1^ scan rate; and 5 accumulations. CD spectra were recorded at 37 °C using a 2-mm cell. The concentration of each enzyme was 1.0 µM. CD spectra were processed with a Jasco software, and finally expressed in mean-residue molar ellipticity units, [*θ*] (deg cm^2^ dmol^−1^).

### Thermal denaturation

WT and variants (1.5 μM) in 100 mM 4-(2-hydroxyethyl)-1-piperazineethanesulfonic acid (HEPES)-NaOH buffer (pH 8.0) were incubated at 37 °C for 5 min. Then, the solution (600 μL) was transferred to a 2-mm cell, which 50 μL of mineral oil was added to avoid evaporation. Thermal denaturation was examined by monitoring the [*θ*] value at 222 nm, [*θ*]_222_, from 25 to 90 °C at 0.5 °C/min. The fraction unfolded (*F*_u_) was determined after normalizing [*θ*]_222_ of native and denatured enzymes between 0 and 1, according to Eq. (1).

1 $$F_{{\text{u}}} = \, \left( {A_{{\text{O}}} - A_{{\text{N}}} } \right)/\left( {A_{{\text{D}}} - A_{{\text{N}}} } \right)$$

where *A*_O_ is the observed [*θ*]_222_ of WT or variants at various temperatures, and *A*_N_ and *A*_D_ are *θ*_222_ of native and denatured enzymes, respectively. The temperature at which *F*_u_ is 0.5 is defined as an apparent denaturing temperature (*T*_m_).

### Thermal inactivation

WT and variants (1.5 μM) in 100 mM HEPES–NaOH buffer (pH 8.0) were incubated at 58–72 °C for 0–60 min. Then, the beechwood xylan-hydrolyzing activity was determined as described above. The first-order rate constant of thermal inactivation (*k*_obs_) was evaluated by plotting natural logarithm of the residual activity against the duration time of thermal treatment. The activation energy for the thermal inactivation (*E*_a_) and the standard Gibbs energy change of activation for thermal inactivation (*ΔG*°^‡^) were determined according to Arrhenius plot (Eqs. 2, 3), respectively.

2 $${\text{ln }}\left( {k_{{{\text{obs}}}} } \right) \, = {-}\left( {E_{{\text{a}}} /R} \right) \, \left( {{1}/T} \right) + {\text{Const}}$$

3 $$\Delta G^{\circ \ddag } = {-}RT\left[ {{\text{ln }}\left( {k_{{{\text{obs}}}} } \right) \, {-}{\text{ ln }}\left( {RT/Nh} \right)} \right]$$

where *R* is the gas constant (= 8.314 J K^−1^ mol^−1^), *T* is absolute temperature in degrees Kelvin, *N* is Avogadro number (= 6.022 × 10^23^ mol^−1^), and *h* is Plank constant (= 6.626 × 10^−34^ J s), respectively. The standard enthalpy change of activation for thermal inactivation (Δ*H*°^‡^) was estimated according to Eq. (4). Using the estimated *ΔG*°^‡^ and *ΔH*°^‡^ values at 65 °C, the standard entropy change of activation for thermal inactivation (*ΔS*°^‡^) was estimated according to . (5).

4 $$\Delta H^{ \circ \ddag } = E_{{\text{a}}} {-}RT$$

5 $$\Delta S^{\circ \ddag } = \, \left( {\Delta H^{ \circ \ddag } {-}\Delta G^{ \circ \ddag } } \right)/T$$

### Supplementary Information

Supplementary Figures.

Supplementary Tables.

## Data Availability

The atomic coordinates and structure factors reported in this study were deposited in the Protein Data Bank under accession code 8XY0 for ligand-free T315Q and 8Y1M for WT complexed with xylobiose. The data sets used and analyzed during the current study are available from the corresponding author upon reasonable request.
