# Supplementary material for: Activity-stability trade-off observed in variants at position 315 of the GH10 xylanase XynR
Source: Sci Rep. 2024 Apr 2;14:7767. doi: 10.1038/s41598-024-57819-z (PMC10987496; doi:10.1038/s41598-024-57819-z)
Supplement: Supplementary file 1 — Supplementary Figures. [file 41598_2024_57819_MOESM1_ESM.pdf]

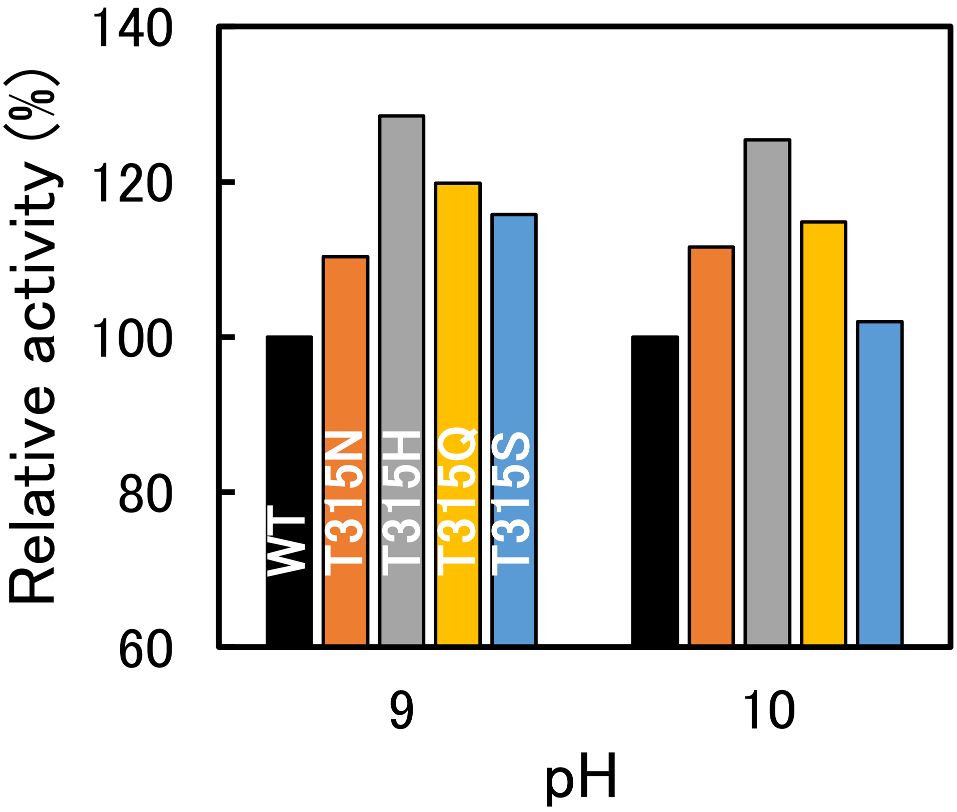

Fig. S1. Activity at pH 9 and 10 of WT and variants. Hydrolysis reaction of beechwood xylan was carried out at 37°C with the enzyme and initial substrate concentrations of 0.5 μM and 9 mg/mL, respectively. The reaction buffers was100 mM carbonate–bicarbonate buffer (pH 9.0–10.0). Relative activity indicates the value compared to that of WT. One of the results of triplicate determinations is shown.

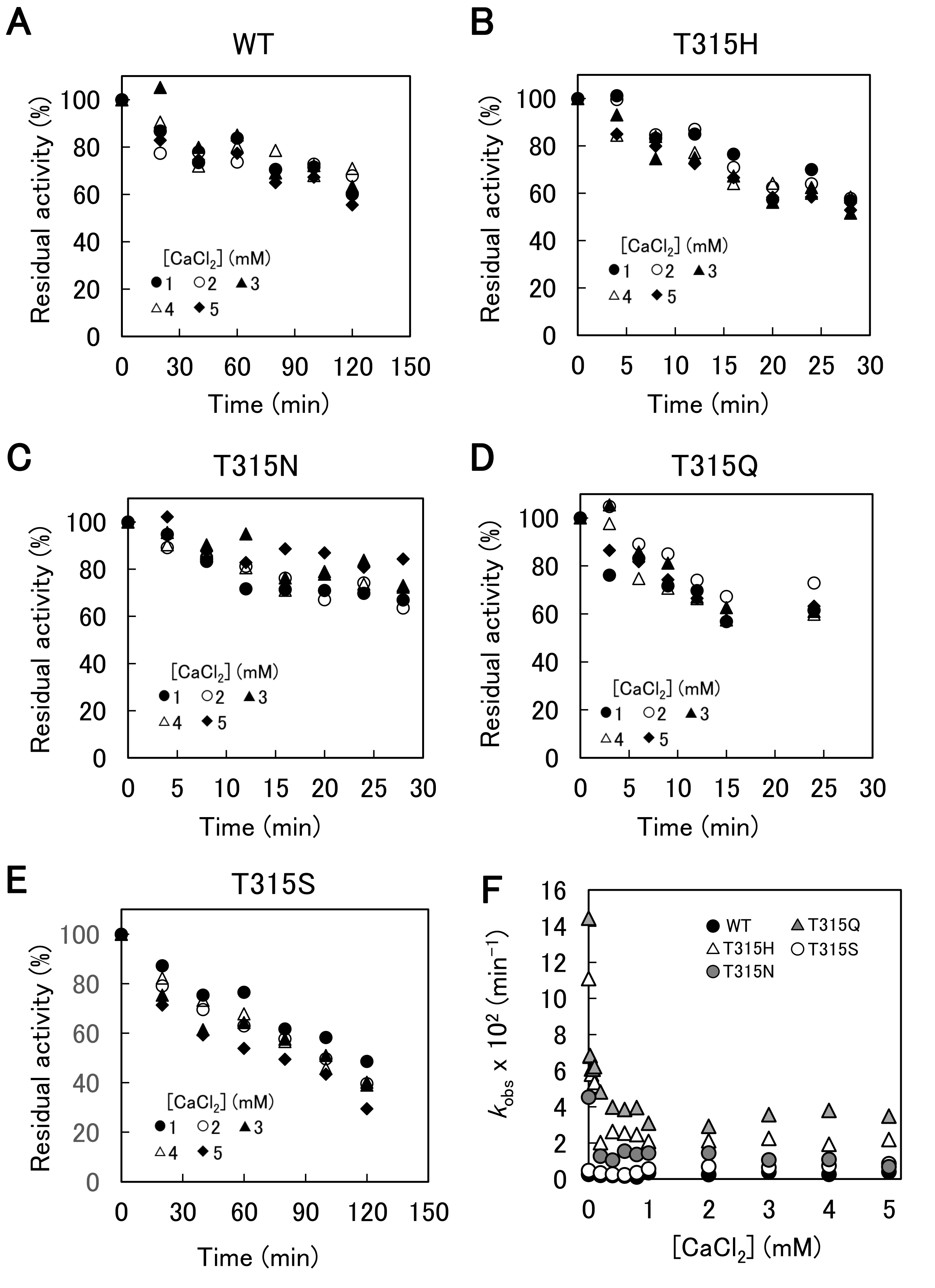

Fig. S2. Effect of $CaCl_2$ concentrations on thermal inactivation of WT and variants. The enzymes (1.5 μM) were incubated in 100 mM HEPES–NaOH buffer (pH 8.0) and 0–5 mM $CaCl_2$ at 62°C for specified time. Beechwood xylan-hydrolysis activities were measured at 37°C after thermal incubation. (A–E) Residual activities of WT and four variants were plotted against incubation time. (F) First-order rate constants of thermal inactivation (k_{obs}) of WT and four variants were plotted against $CaCl_2$ concentrations of 0–5 mM.

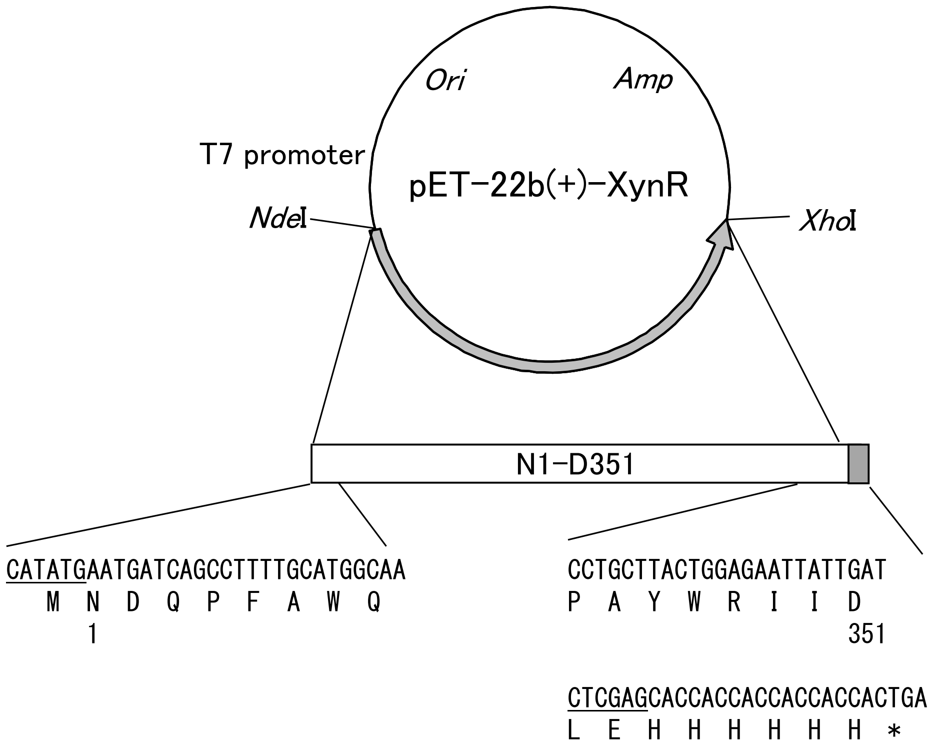

Fig. S3. Expression plasmid for XynR. The asterisk indicates the termination codon. The *Nde*I and *Xho*I sites are underlined.
