# Supplementary material for: Activity-stability trade-off observed in variants at position 315 of the GH10 xylanase XynR
Source: Sci Rep. 2024 Apr 2;14:7767. doi: 10.1038/s41598-024-57819-z (PMC10987496; doi:10.1038/s41598-024-57819-z)
Supplement: Supplementary file 2 — Supplementary Tables. [file 41598_2024_57819_MOESM2_ESM.docx]

**Table S1.** First-order rate constants (*k*_obs_) and half-lives (*τ*_1/2_) of thermal inactivation of WT and variants

*k*_obs_ × 10^3^ (min^-1^)

Temperature (ºC) 58 59 60 61 62 63 64 65 66 67 68 69 70 71 72

WT 24 20 50 70 120 120

T315H 19 31 47 65 120

T315N 40 53 88 99 120 220

T315Q 23 31 51 67 140 210

T315S 　　　　　　　　　　　　29 34 43 110 81 120

*τ*_1/2_ (min)

Temperature (ºC) 58 59 60 61 62 63 64 65 66 67 68 69 70 71 72

WT 29 36 14 10 5.8 5.9

T315H 36 22 15 11 5.9 　3.3

T315N 17 13 7.8 7.0 5.7 3.2

T315Q 30 22 13 10 4.8 3.2

T315S 　　　　　　　　　　　　24 20 16 6.4 8.5 5.7

**Table S2.** First-order rate constants (*k*_obs_) and half-lives (*τ*_1/2_) of thermal inactivation of WT and variants at 62ºC

*k*_obs_ × 10^3^ (min^-1^)

CaCl_2_ (mM) 0 0.025 0.05 0.075 0.1 0.2 0.4 0.6 0.8 1.0 2.0 3.0 4.0 5.0

WT 2.6 2.1 　2.0 1.7 1.2 3.5 2.5 4.0 2.7 4.1

T315H 110 68 58 52 54 20 26 26 25 21 21 23 19 22

T315N 45 13 11 16 14 14 14 11 11 7.0

T315Q 140 68 61 61 62 48 40 39 40 31 29 36 39 35

T315S 4.8 3.4 2.8 2.4 3.6 5.7 7.0 6.5 7.4 8.6

*τ*_1/2_ (min)

CaCl_2_ (mM) 0 0.025 0.05 0.075 0.1 0.2 0.4 0.6 0.8 1.0 2.0 3.0 4.0 5.0

WT >100 >100 　>100　>100　>100　>100 >100　>100　 >100　 >100

T315H 6 10 12 13 13 34 26 27 28 33 33 31 36 32

T315N 15 55 66 45 51 48 48 65 64 100

T315Q 5 10 11 11 11 14 17 18 18 22 24 19 18 20

T315S >100 >100 　>100　>100　>100　>100 >100　>100　 >100　 >100
